# Upsizing of GORE® Cardioform ASD Occluder for Atrial Septal Defect With Atrial Septal Aneurysm

**DOI:** 10.7759/cureus.63281

**Published:** 2024-06-27

**Authors:** Hiromasa Hayama, Kenji Makino, Yoshiyuki Yazaki, Hidehiko Hara

**Affiliations:** 1 Division of Cardiovascular Medicine, Toho University Ohashi Medical Center, Tokyo, JPN

**Keywords:** echocardiography, gore cardioform septal occluder, pfo, asa, asd

## Abstract

Atrial septal defects (ASDs) often present with multiple foramina, including a patent foramen ovale (PFO) and atrial septal aneurysms (ASAs). Transcatheter device closure of an ASD may require additional supportive techniques in complex cases. Here, we report a case of a secundum ASD complicated by an ASA and a PFO in a man in his 50s. A GORE® Cardioform ASD Occluder (GCA) device of the optimal size for balloon sizing was implanted. However, edge leakage occurred from the front of the device because of a large, moving ASA. Implantation of a two-size-up GCA device successfully closed the ASD under controlled ASA movement.

## Introduction

Atrial septal defects (ASDs) are the most common congenital heart disease and are characterized by the absence of tissue at the atrial septum. ASDs can be categorized into several types: ostium secundum, ostium primum, sinus venosus, and coronary sinus defects. The most common type is the ostium secundum, which accounts for about 70-75% of all ASDs [1]. Untreated ASDs may lead to right ventricular volume overload, atrial arrhythmias, and pulmonary arterial hypertension [2]. There are various types of transcatheter closure devices for ASD, and the treatment depends on the length of the rim around the ASD and the number of defects [3]. The GORE® Cardioform ASD Occluder (GCA) has recently been used as an excellent closure device for patients with a rim defect and a large ASD. The GCA consists of a nitinol wire frame filled with platinum and covered with expanded polytetrafluoroethylene, making it softer and more conformable compared to other devices [4]. The GCA has achieved high technical success and closure rates while maintaining a low incidence of severe adverse events and device-related complications. It has been shown to be effective for patients of a wide age range, regardless of the presence of an aortic rim defect. Additionally, the high number of successful post-procedural implants and the absence of aortic erosion underscore the importance of the GCA device for interventionists who close ASDs [5]. However, the actual use of GCA is still limited worldwide. We report that in more complex cases, such as those involving atrial septal aneurysm (ASA), it was necessary to upsize the GCA beyond the recommended size.

## Case presentation

**Figure 1:**
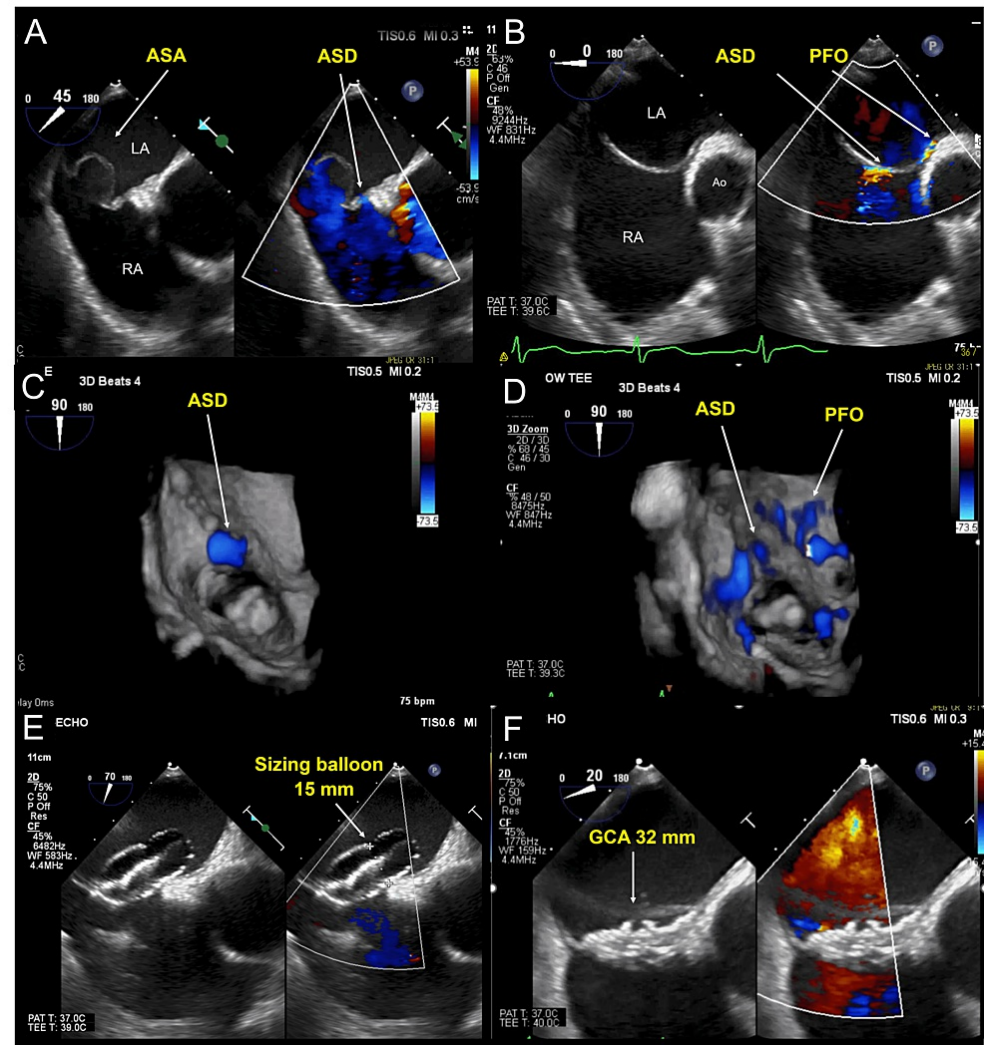
TEE imaging for measurement in ASD, PFO, and ASA and deployment of a 32-mm GCA device (A) TEE showed a partial anterior rim defect and floppy ASA in the posterior rim in both directions of the left and right atrium. (B) A PFO was also present. (C and D) ASD and PFO by 3D TEE. (E) The sizing balloon at stop-flow through the ASD was 15 mm. (F) A 32-mm GCA was implanted based on the sizing balloon calculation. 3D, three-dimensional; ASA, atrial septal aneurysm; ASD, atrial septal defect; GCA, GORE® Cardioform ASD Occluder; PFO, patent foramen ovale; TEE, transesophageal echocardiography

A man in his 50s was referred for ASD closure because of a secondary foramen defect type ASD noted at the age of 20 years. In addition to ASD, transthoracic echocardiography showed right ventricular enlargement. In addition, he had left lower extremity venous thrombosis and was taking anticoagulants. Transesophageal echocardiography (TEE) showed an ASD size of 8.6 × 6.3 mm with a partial anterior rim defect and a floppy ASA in the posterior rim in both directions of the left and right atrium, with the ASA mobilizing over 15 mm (Figure 1A). Moreover, a patent foramen ovale (PFO) was also present (Figure 1B, 1C, 1D), and the distance between the ASD and PFO was 8 mm. We selected the GCA since the device uses a softer material and is larger and safer to cover than the other devices. The size of the GCA was critical for this patient because we intended to close the ASD and PFO.

Procedure details

**Figure 2:**
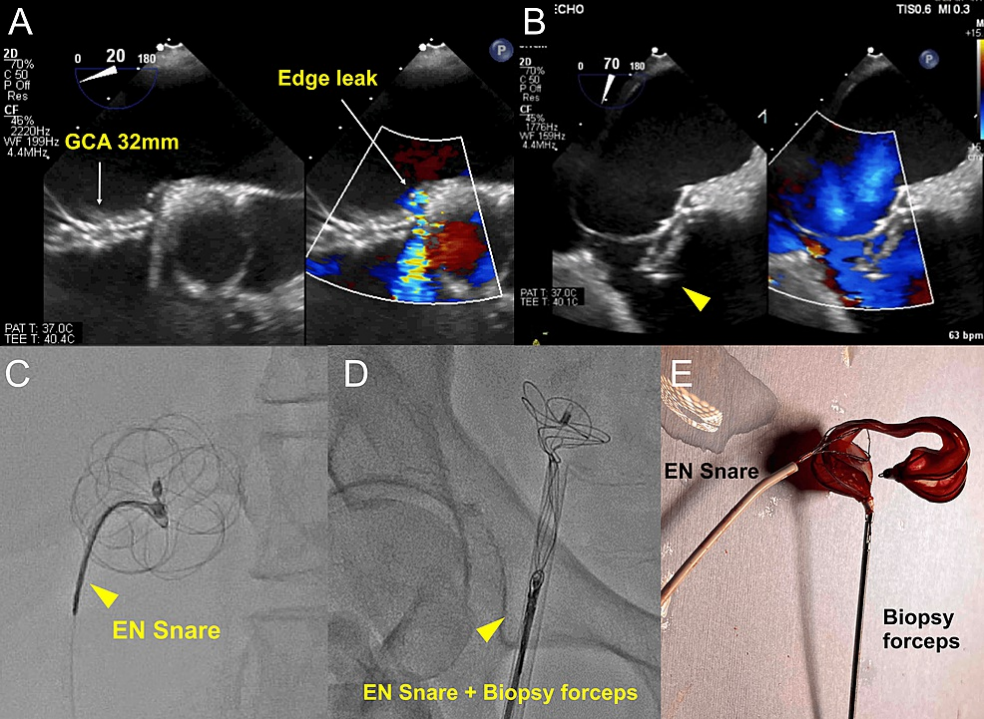
Leakage and retrieval after GCA device deployment (A) Edge leakage occurred from the front of the GCA device. (B) The rear of the device dislodged. (C) The EN Snare® endovascular snare (12-20 mm) grasped the central portion of the GCA. (D and E) Combined with the snare and biopsy forceps, the device was retrieved. GCA, GORE® Cardioform ASD Occluder

**Figure 3:**
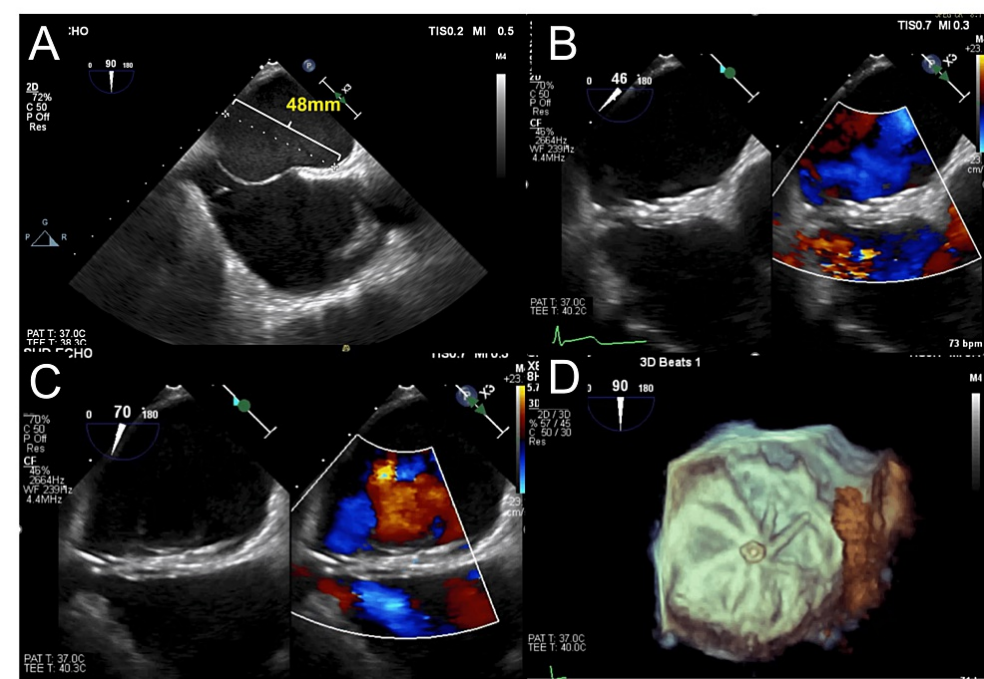
Insertion of an upsized GCA device (A) The left atrial diameter was 48 mm, allowing the insertion of an even larger GCA device. (B and C) A 44-mm GCA was implanted to cover the ASD, ASA, and PFO and to control the movement of the ASA. (D) 3D TEE image after the 44-mm GCA device implantation. 3D, three-dimensional; ASA, atrial septal aneurysm; ASD, atrial septal defect; GCA, GORE® Cardioform ASD Occluder; PFO, patent foramen ovale; TEE, transesophageal echocardiography

**Figure 4:**
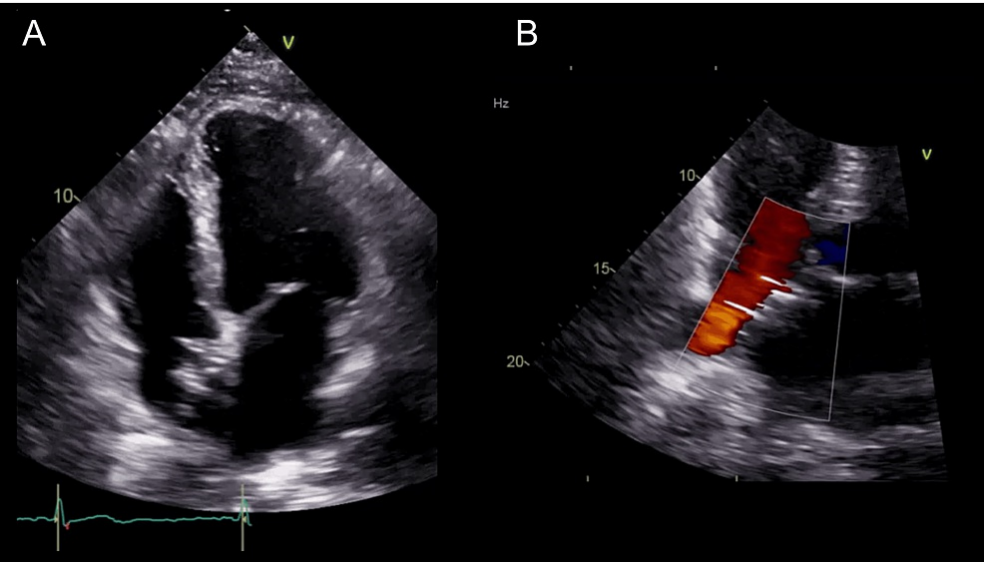
Postoperative TEE imaging (A) Postoperatively, there was no dislodgement of the 44-mm GCA. (B) No residual shunt after placement of the 44-mm GCA. GCA, GORE® Cardioform ASD Occluder; TEE, transesophageal echocardiography

A 10 French (Fr) sheath was used to approach the right femoral vein. The wiring to the ASD was performed under TEE guidance. The sizing balloon (Amplatzer Sizing Balloon, Abbott Plymouth, USA) was measured through ASD and was 15 mm in diameter at stop-flow on TEE evaluation (Figure 1E). Consequently, we chose to implant a 32-mm GCA (Figure 1F). However, after releasing the GCA device, the floppy behavior of the ASA caused the device to gradually move backward, resulting in front-edge leakage (Figure 2A). The leakage originates from the front of the GCA device within the ASD. In addition to this, the rear of the device dislodged (Figure 2B). For percutaneous GCA device retrieval, a 5 Fr Judkins right coronary catheter with an EN Snare® Endovascular Snare (12-20 mm) was used to grasp the protrusion at the center of the GCA (Figure 2C). The operator attempted to pull the device into the 14 Fr sheath; however, the device was so large that it detached from the snare in front of the sheath. Biopsy forceps grasped the protrusion at the center of GCA, and the snare was repositioned to capture the lower part where the device folds during retraction for retrieval (Figure 2D). Both instruments were simultaneously used to retrieve the device (Figure 2E). The atrial diameter measured by TEE was 48 mm, and the insertion of a larger device was adequate (Figure 3A). Therefore, a 44-mm GCA device (two sizes larger) was implanted to successfully close the ASD with controlled movement of the ASA (Figure 3B, 3C, 3D). Postoperative echocardiography revealed no dislodgement of the GCA or residual shunt (Figure 4A, 4B).

## Discussion

Device selection

Our study highlights the importance of correct device selection for ASD closure, particularly in cases involving ASAs and PFOs. The GCA device, a relatively new ASD closure device, is known for its ability to safely occlude large ASDs, especially in patients with rim defects [4]. Despite multiple device options, the choice of the GCA was based on its specific advantages. For instance, the Amplatzer Septal Occluder (ASO) (Abbott, St. Paul, USA) has a strong grasping force and is often chosen for ASD closure with ASA [6]. However, an ASO device has a risk of erosion to the aorta due to the patient’s defect in the anterior rim. The Occlutech’s Figulla Flex II ASD Occluder (FSO) (Occlutech International AB, Helsingborg, Sweden) is effective for anterior rim defects and could be an alternative to GCA, but we considered the GCA to be softer and safer in closing the ASD than the FSO.

Management of PFO

Another important factor in this case was the presence of PFO in this patient. PFO associated with ASD is not treated aggressively in most cases because of its minimal impact on the volume-loading effect of PFO shunts. In recent years, PFO closure by percutaneous catheter has been actively performed in stroke patients due to the risk of paradoxical cerebral embolism caused by a venous thrombus in the lower extremities [7]. Although this patient had no history of stroke, he suffered from a left lower extremity venous thrombus. Therefore, we aimed to cover the PFO with the device by placing the GCA on the aorta in an A shape. In this case, a strategy was devised with the primary objective of treating the ASD due to the observation of right heart overload as a result of the ASD shunt. The ASD could not be effectively controlled with the PFO device. Furthermore, since there was no history of paradoxical embolism, using a PFO device was not an option.

Challenges with ASA

One of the main challenges in this case was the presence of ASA. Prospective case-control studies have reported the presence of ASD in 2.3% of ASA patients [8]. The GCA, designed to softly grasp the ASD rim, initially appeared to be securely fixed by the locking system. However, the significant movement of the ASA resulted in the posterior inferior part slipping off due to the ASA movement. The dislodgement of the GCA in this case is strongly attributed to the influence of the ASA. Initially, after placement, the ASD and PFO were controlled. However, the movement of the ASA caused the GCA to dislodge posteriorly, followed by an anterior edge leak. Given the GCA device’s design, which is to softly grasp the ASD rim, there was a risk of it slipping off when the rim was in motion. Furthermore, the GCA does not have strong grasping power on the rims and may not be adaptable in normal size to the ASA with excessive motion. Therefore, in this case, the GCA became dislodged.

Technical considerations

We retrieved the GCA percutaneously and found that using a double-tool retrieval technique was effective for large devices such as the GCA. We recommend using one tool to stabilize or elongate the device and the other tool to pull it back into the sheath. A similar technique was reported in a previous report, where a 30-mm Gore Cardioform device was retrieved using a two-device technique (gooseneck snare and grasping forceps) to retrieve the device into the sheath [9]. As in this case, when the ASA has significant mobility, there is a possibility that the device may become dislodged. The movement of the ASA was considerable, and the length from the posterior wall of the left atrium to the base of the anterior rim was 48 mm. During the procedure, we discussed that controlling the movement of the ASA was crucial, and therefore, inserting a device at the maximum appropriate size was necessary. For this particular patient, the use of a GCA that was two sizes larger than the recommended size was critical in ensuring closure of the ASD under the circumstances of ASA movement. While the ASO has been the most used device historically, the presence of an ASA has been reported to increase the risk of erosion [10]. Although the FSO is considered a treatment option, it may be necessary to upsize the device beyond the standard size, as demonstrated in this case. Ultimately, the upsized GCA fit securely immediately after the procedure without dislodgement. Considering the floppy nature of the ASA, it is highly probable that the larger device was able to adapt appropriately. Key learning points from this case include the importance of anticipating ASA behavior and considering device upsizing preemptively.

## Conclusions

In the percutaneous closure of ASD with ASA, the standard size of the GCA, determined by balloon sizing, may carry a risk of dislodgement, particularly in cases involving significant ASA movement. To fully control the ASA and ensure successful closure, it is advisable to use a device larger than the recommended size. Additionally, during the retrieval of the GCA device, employing a dual-tool technique with instruments like snares and biopsy forceps has proven effective. This case showed no adverse events immediately post-procedure, despite using a larger GCA device. It highlights that in ASD cases with an anterior rim defect and excessively mobile ASA, a soft GCA is a good option, but sufficient upsizing is necessary. Future research is needed to validate these findings and explore the efficacy and safety of various ASD closure devices in complex cases involving ASAs and PFOs.
